# Nicotinamide Improves Skin Photoaging in Mice by Delaying Cellular Senescence and Suppressing the Senescence-Associated Secretory Phenotype

**DOI:** 10.3390/cimb48070661

**Published:** 2026-06-27

**Authors:** Xin-Yue Tang, Ke-Jin Lu, Rui Zhu, Yue Gao, Dong-Yan Wei, Xi-Yu Zhang, Yi-Cheng Ma, Fei-Fei Wang, Cheng-Gang Zou

**Affiliations:** 1 State Key Laboratory for Conservation and Utilization of Bio-Resources in Yunnan and Yunnan Key Laboratory of Basic Research and Innovative Application for Green Biological Production, School of Life Sciences, Southwest United Graduate School, Yunnan University, Kunming 650500, China; tangxinyue1130@163.com (X.-Y.T.); lqldh@hotmail.com (K.-J.L.); zhuzhurui2021@163.com (R.Z.); weidy1245@163.com (D.-Y.W.); sigar0524@163.com (X.-Y.Z.); mayc@ynu.edu.cn (Y.-C.M.); 2 Yunnan Characteristic Plant Extraction Laboratory, Kunming 650106, China; gy2020cpu@163.com

**Keywords:** nicotinamide, skin photoaging, senescence-associated secretory phenotype, senescence marker

## Abstract

Nicotinamide (NAM), a precursor of nicotinamide adenine dinucleotide (NAD^+^), and NAD^+^ are integral to a variety of cellular processes. NAM supplementation has been shown to have benefits for cellular senescence. However, the mechanism by which NAM improves skin photoaging remains unclear. In this study, the multi-omics analysis revealed that insufficient nicotinamide metabolism may be associated with a decrease in NAD^+^ synthesis during skin aging. Importantly, we found that NAM has an ameliorating effect on the skin photoaging in mice. Supplementation with NAM restored the expression of the salvage-pathway enzymes and NAD^+^ consumers. In addition, the supplementation with NAM was shown to restore the expression of skin barrier-related proteins (ZO1 and E-cadherin) and collagen I, while reducing the expression of senescence markers (γ-H2AX, p53, and p21). Furthermore, we found that NAM effectively suppresses the senescence-associated secretory phenotype (SASP) factors’ expression in skin photoaging. Our research reveals the dual role of NAM in attenuating skin photoaging, acting not only to delay cellular senescence but also to suppress the SASP.

## 1. Introduction

The term skin “photoaging” is defined as the process of skin aging caused by prolonged exposure to solar radiation, encompassing ultraviolet (UV), visible, and infrared wavelengths [1]. These exposures can lead to structural damage and loss of function in the epidermis, dermis, and subcutaneous tissue [1,2]. The typical pathological features of skin photoaging include wrinkles, hyperpigmentation, hyperkeratosis, capillary dilation, barrier damage, and loss of elasticity [2]. Sunlight has been shown to induce direct DNA damage and reactive oxygen species (ROS), which in turn amplify cellular damage through photoreactions and lead to photoaging [3]. Although, the regulatory mechanisms of skin photoaging have been elucidated to some extent, including epigenetic alterations and the activation of related signaling pathways (e.g., MAPK and NF-κB), as well as the senescence-associated secretory phenotype (SASP) and the breakdown of the extracellular matrix (ECM) [1,4,5]. However, the involvement of certain endogenous metabolites in skin photoaging has not yet been reported.

Nicotinamide adenine dinucleotide (NAD^+^) levels decline progressively in multiple tissues during aging, and this ubiquitous depletion has been increasingly recognized as a central metabolic hallmark that couples diverse aspects of the aging process [6,7,8]. NAD^+^ biosynthesis in mammals proceeds through three principal routes: the salvage pathway, the de novo synthesis pathway and the Preiss–Handler pathway [9]. The salvage pathway and the Preiss–Handler pathway use different forms of vitamin B3 to produce NAD^+^. The salvage pathway uses nicotinamide (NAM) and nicotinamide nucleoside (NR), while the Preiss–Handler pathway uses nicotinic acid (NA) [10]. In mammals, the primary de novo synthesis pathway is the tryptophan–kynurenine pathway, whereby NAD^+^ is synthesized from tryptophan [11].

NAM acts as a precursor of nicotinamide adenine dinucleotide (NAD^+^) and its phosphate derivative (NADPH) [12]. These cofactors and their reduced forms (NADH and NADPH) serve as coenzymes in many redox reactions [12]. In ALD (alcohol-associated liver disease) mouse, NAM exhibited a robust capacity to suppress senescence in liver cells, reducing liver injury markers and improving metabolic function [13]. NAM has also been shown to extend the replication lifespan of normal human fibroblasts [14]. In addition, NAM blocks the production of ROS in cells, preventing oxidative damage to DNA and cell membrane lipids and thus the defective cellular functioning that becomes visible as skin aging [15]. Topical NAM has been shown to readily penetrate human skin and increase local NADH levels [16]. Furthermore, several clinical studies have shown a significant reduction in age-related yellowing of the skin, caused by the Maillard reaction (spontaneous oxidation and glycation of proteins), after the application of topical NAM [17,18,19]. However, the regulatory mechanisms by which NAM ameliorates skin photoaging remain unclear.

Previous studies have shown that NAM prevents photoaging in both two-dimensional human primary keratinocyte cultures and three-dimensional organotypic epidermal models [20]. In the present study, it was demonstrated that supplementation with NAM has the potential to ameliorate skin photoaging in mice, with this effect attributed to delaying cellular senescence and suppressing the expression of SASP factors. Consequently, our research has revealed the dual role of NAM in counteracting photoaging in mice.

## 2. Materials and Methods

### 2.1. Statement of Ethics

The Animal Care and Use Committee of Yunnan University approved the procedures for mice studies, care and maintenance (Approval No. YNU20240708, approval date: 11 January 2024). The investigation of our protocol conforms to the Guide for the Care and Use of Laboratory Animals, published by the US National Institutes of Health (NIH).

### 2.2. Animal Models

Female Balb/c mice (6–8 weeks of age, weight range at 19 ± 1 g) were obtained from the Henan Skobes Biotechnology Co., Ltd. (Huaxian, China). The mice were maintained at a constant temperature of 25 ± 2 °C, humidity levels of 60 ± 5%, with a 12 h light/12 h dark cycle and free access to food and water. All of the mice used in the experiment had their dorsal hair removed with a razor 2 days before the experiment began. The UV light source used a solar simulator SUV1000 (SIGMA High-tech Co., Shanghai, China), with an initial irradiation dose of 1 minimal erythema dose (MED) (70 mJ/cm^2^) for mice, increasing by 0.5 MED weekly for a total of 9 weeks, with irradiation 5 days per week. The intensity of UV exposure varied was shown in Table S1. The SUV1000 was equipped with a UV(A + B) filter, producing a spectrum of approximately 95% UVA and 5% UVB, which simulates natural sunlight. All mice were randomly divided into 6 groups, with 6 mice in each group: (1) normal mice (Con), (2) photoaging mice (UV), (3) photoaging mice treatment with 200 μL 2% (*w*/*v*) NAM (Nicotinamide, MACKLIN) (2), (4) photoaging mice treatment with 200 μL 5% (*w*/*v*) NAM (5), (5) photoaging mice treatment with 200 μL 7.5% (*w*/*v*) NAM (7.5), and (6) photoaging mice treatment with 200 μL 12.5% (*w*/*v*) NAM (12.5). In this study, The NAM treatment was initiated at the beginning of the third week of the 9-week UV irradiation period. Therefore, the total duration of NAM treatment was 7 weeks. NAM was applied topically immediately after UV exposure, once daily, five times per week.

### 2.3. Evaluation of Photoaging

At the conclusion of the experiment, in accordance with the scoring criteria established by Zheng et al. [21], 3 observers conducted a comprehensive evaluation of the dorsal skin condition of the mice. The grading score ranged from 0 for normal skin to 6 for severely damaged skin. The photoaging score was shown in the Table S2.

### 2.4. Skin Tissue Staining

Skin tissues were harvested, with aliquots immediately snap-frozen in liquid nitrogen for subsequent RNA extraction and gene expression analysis. The remaining tissue portions were fixed in 4% paraformaldehyde, dehydrated through a graded ethanol series, and embedded in paraffin. Sections (4 μm) were cut and stained with hematoxylin and eosin (H&E) according to standard protocols. Images were acquired using a Zeiss Axioskop 2 plus fluorescence microscope (Carl Zeiss, Jena, Germany), and epidermal thickness was quantified using ImageJ (version 1.53c, NIH, Bethesda, MD, USA).

### 2.5. Western Blotting

20 mg skin tissues were homogenized in liquid nitrogen, and then resuspended in RIPA buffer (Solarbio, Beijing, China) on ice for 30 min. Lysates were clarified by centrifugation at 12,000× *g* for 15 min at 4 °C. A total of protein lysates (20–50 μg) per sample was loaded, and separated onto 10% to 12% SDS-PAGE gel. The primary antibodies used were anti-p21 antibody (ab188224, Abcam, Waltham, MA, USA), anti-p53 antibody (TA0865S, Abmart, Shanghai, China), anti-Collagen I antibody (ab255809, Abcam, Waltham, MA, USA), anti-ZO1 antibody (21773-1-AP, Proteintech, Wuhan, China), anti-E-cadherin antibody (20874-1-AP, Proteintech, Wuhan, China), anti-Phospho-Histone H2AX antibody (AP0687, ABclonal, Wuhan, China), anti-MMP12 antibody (A1709, ABclonal, Wuhan, China), anti-MMP1 antibody (10371-2-AP, Proteintech, Wuhan, China), anti-Phospho-TFEB antibody (87932S, CST, Danvers, MA, USA) and anti-GAPDH antibody (ab8245, Abcam, Waltham, MA, USA). The secondary antibody was a Multi-rAb™ HRP-Goat Anti-Rabbit Recombinant Secondary Antibody (H + L) (RGAR001, Proteintech, Wuhan, China) or Goat Anti-Mouse IgG, HRP Conjugated (CW0102S, CoWin Bioscience, Jiangsu, China). Proteins were then electroblotted onto PVDF membranes (Merck Millipore Corp., Darmstadt, Germany). The membrane was exposed to Thermo Scientific Pierce ECL Western blotting substrate (Thermo Fisher Scientific, Waltham, MA, USA). A GE Amersham Imager 600 imaging system (GE Healthcare, Pittsburgh, PA, USA) was used for detection of the blot results. Subsequent image analysis was performed using ImageJ software (NIH). Results were from triplicate experiments. Uncropped images of Western blots are shown in Figures S1–S3.

### 2.6. Immunohistochemical Analysis

Paraformaldehyde-fixed paraffin-embedded skin sections were subjected to antigen retrieval using a Tris-EDTA antigen retrieval solution (pH 9.0) (Servicebio, Beijing, China). Then, the sections were blocked with 3% BSA at room temperature for 30 min. The sections were stained with anti-p21 antibodies (ab188224, Abcam, Waltham, MA, USA) overnight at 4 °C and then the sections were washed with PBS three times at room temperature. Then the sections were incubated with HRP conjugated Goat Anti-Rabbit IgG (GB23303, Servicebio, Wuhan, China) at room temperature for 1 h. Then the substrate solution diaminobenzidine (G1212, Servicebio, Wuhan, China) was added into the sections for color development. Then the sections were counterstained with hematoxylin. Images were acquired using a Zeiss Axioskop 2 plus fluorescence microscope (Carl Zeiss, Oberkochen, Germany). ImageJ software was used to determine the average number of positive cells, average density, and average optical density.

### 2.7. Real-Time Quantitative PCR

20 mg skin tissues were homogenized in liquid nitrogen, and then resuspended in 1 mL of Trizol Universal Reagent (DP424, Tiangen, Beijing, China). Then add 200 μL of RNA extraction auxiliary reagent (es-8522, Ecotopbio, Guangzhou, China) to extract total RNA. cDNA was generated using a first strand cDNA synthesis kit (KR118, Tiangen). SYBR Green (FP217, Tiangen) and Thermo Fisher Scientific, QuantStudio™ 3 System (Thermo Fisher Scientific) were utilized for qPCR. 2^−ΔΔCT^ method was used to calculate relative expression normalized to an internal control of mice *Gapdh*. All primers were synthesized by Beijing Tsingke Biotechnology Co., Ltd. (Beijing, China), and the sequence of primers was shown in Table S3.

### 2.8. Dataset Collection

We collected two data sets of mouse skin tissues, including GSE296578 and GSE279221. The GSE296578 data contained whole-genome RNA sequencing data from skin tissues of chronic photoaging mice and normal controls. The GSE279221 data contained whole-genome RNA sequencing data from skin tissues of acute photoaging mice and normal controls. All of the above data sets are from GEO public database (https://www.ncbi.nlm.nih.gov/geo/ accessed data: 22 July 2025). For single-cell transcriptomic data, we employed GSE274955, the project that contains raw single-cell RNA sequencing data from arm and back skin samples from healthy adults of different ages.

### 2.9. Functional Enrichment Analysis

The *p*-values ≤ 0.05 and a fold change ≥ 1.5 were used as the threshold values for differential expression. For the Gene Ontology enrichment analysis of SASP, we searched for the enrichment of the Kyoto Encyclopedia of Genes and Genomes (KEGG) pathway gene sets of the transcriptomic data sets of the skin tissues using Gene Set Enrichment Analysis (GSEA). The GSEA was built based on the GSEA R toolkit (R v4.3.3, GSEA v2.1) [22].

### 2.10. scRNA-Seq Data Analysis

Single-cell RNA sequencing (scRNA-seq) data from GSE274955 were processed using the Seurat R package (v5.2.1). Prior to downstream analysis, cells were filtered according to published quality metrics [23], removing those with <200 or >6000 detectable genes or a mitochondrial read fraction exceeding 10%. Normalization and variance-stabilizing transformations were carried out with SCTransform. For clustering, we computed the first 30 principal components and fed them into the FindClusters function (resolution 0.8). The resulting high-dimensional structure was projected onto two dimensions via UMAP (RunUMAP) for graphical display. To identify cluster-specific transcriptional signatures, we applied the FindAllMarkers function (Wilcoxon rank-sum test), retaining genes that met log_2_FC > 0.25 and adjusted *p* < 0.05. Cell annotation was performed by the first 30 genes in each cluster.

### 2.11. Statistical Analysis

Statistical analyses were performed using GraphPad Prism (version 8). All data are presented as the mean ± SD. The significance of differences was evaluated by Student’s *t*-test between groups or by one-way ANOVA followed by the multiple-comparison test. Statistically significant results were defined as *p* ≤ 0.05.

## 3. Results

### 3.1. The Synthesis of NAD^+^ Decreases During Skin Aging

**Figure 1:**
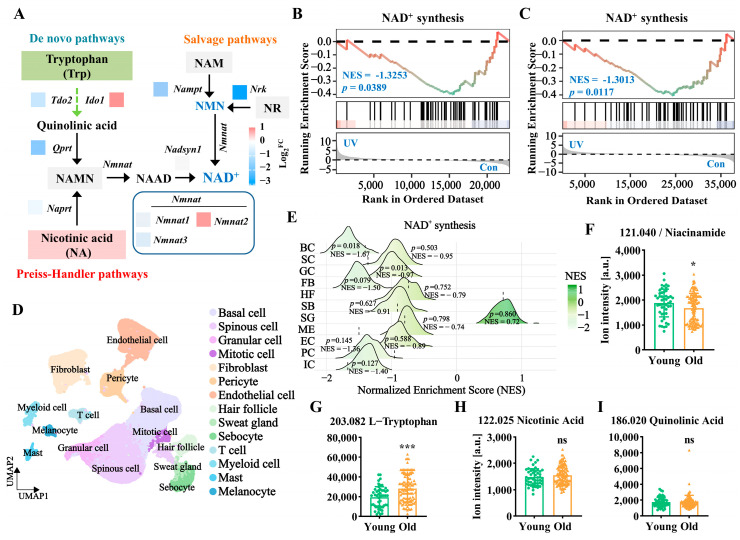
NAD^+^ synthesis-related genes are down-regulated during skin aging. (**A**) The heat map illustrates the RNA-seq analysis results of NAD^+^ synthesis-related genes in skin photoaging (GSE296578). (**B**,**C**) GSEA of NAD^+^ synthesis metabolism in the transcriptional profiles of the GSE296578 (**B**) and GSE279221 (**C**) datasets of skin photoaging mice (UV) versus normal mice (Con). (**D**) Uniform manifold approximation and projection (UMAP) plot showing the cell types of human skin (GSE274955). (**E**) GSEA of NAD^+^ synthesis metabolism in healthy human adult skin samples across various ages, as determined by scRNA-seq (GSE274955). From top to bottom, the GSEA results of BC, SC, GC, FB, HF, SB, SG, ME, EC, PC and IC. BC: basal cell; SC: spinous cell; GC, granular cell; FB, fibroblast; HF, hair follicle; SB, Sebocyte; SG, Sweat gland; ME: melanocyte; EC, endothelial cell; PC, pericyte; IC, immune cell. (**F**–**I**) Differential analysis of Nicotinamide (**F**), L-tryptophan (**G**), nicotinic acid (**H**), and quinolinic acid (**I**) metabolic levels comparing young and old skin. The number in the title reports the measured *m*/*z*. * *p* < 0.05, *** *p* < 0.001, ns, non-significant. *p* values were calculated using a two-tailed unpaired *t*-test.

To investigate the mRNA changes in genes involved in NAD^+^ synthesis in skin photoaging, we first analyzed the RNA-seq data from the GSE296578 dataset. We found that the mRNA levels of NAD^+^ synthesis-related genes including *Tdo2*, *Qprt*, *Nampt*, and *Nrk*, were reduced in the skin of photoaging mice (Figure 1A). Gene Set Enrichment Analysis (GSEA) revealed that NAD^+^ synthesis was significantly downregulated in the transcriptional profiles (GSE296578) of skin photoaging mice compared to normal mice (NES = −1.3253, *p* = 0.0389) (Figure 1B). Meanwhile, the GSEA indicated that the transcriptional profiles of the GSE279221 dataset showed significant downregulation of NAD^+^ synthesis (NES = −1.3013, *p* = 0.0117) (Figure 1C).

Next, we used scRNA-seq (GSE274955) to examine the expression of genes involved in NAD^+^ synthesis in human skin cells, including those from young, middle-aged, and old donors. UMAP visualization revealed that the skin tissues of different ages was divided into 14 cell types, namely basal cell (BC), spinous cell (SC), granular cell (GC), mitotic cell, fibroblasts (FBs), pericyte (PC), endothelial cells (ECs), hair follicle (HF), sweat gland (SG), sebocyte (SB), immune cell (IC, including T cell, Myeloid cell, Mast), and melanocytes (MEs) (Figure 1D). By GSEA, we found that the enrichment of NAD^+^ synthesis was suppressed in basal cells (NES = −1.67, *p* = 0.018) of old donors (Figure 1E).

To further explore the changes in metabolites within the NAD^+^ synthesis pathway during skin aging, we used the untargeted metabolomics data published by Kuehne A et al. [24], which included epidermal skin tissue samples from the medial forearms of 28 young (20–25 years) and 54 elderly (55–66 years) female donors. Metabolites of NAD^+^ synthesis, including L-tryptophan, NA, quinolinic acid (QA) and NAM, were identified in the metabolome. The univariate analysis indicated that the levels of NAM were lower in old donors compared to young donors, while L-tryptophan increased (Figure 1F,G). NA and QA did not change in either population (Figure 1H,I). Taken together, these results suggest that insufficient NAM metabolism may be associated with a decrease in NAD^+^ synthesis during skin aging.

### 3.2. NAM Ameliorates Skin Photoaging of Mice

**Figure 2:**
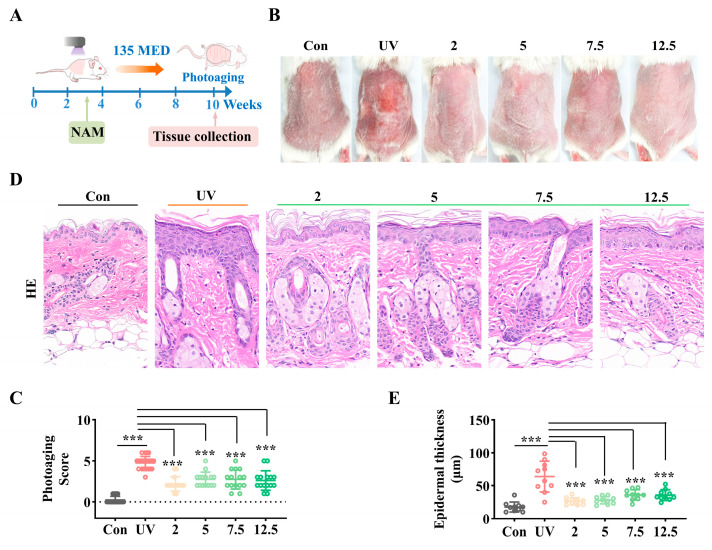
NAM exhibits an effect of anti-photoaging in mice. (**A**) Schematic of the process of establishing a skin photoaging model of female BALB/c mice and the supplementation of NAM. (**B**) Supplementation with NAM has an effective measure in the amelioration of erythema, wrinkles, and signs of extensive flaking in photoaging mice. (**C**) The photoaging scores of mice at 9 weeks (n = 6). These results are means ± SD of three experiments. *** *p* < 0.001. (**D**) Representative images of Hematoxylin-eosin (HE) staining of histological sections among Con, UV, UV + 2% (*w*/*v*) NAM, UV + 5% (*w*/*v*) NAM, UV + 7.5% (*w*/*v*) NAM, and UV + 12.5% (*w*/*v*) NAM. (**E**) Quantification of the epidermal thickness. These results are means ± SD of three experiments. *** *p* < 0.001. *p* values (**C**,**E**) were calculated using one-way ANOVA followed by multiple-comparison test in multiple groups.

To test the effect of NAM on skin photoaging, we developed a model of photoaging using 6-week-old female BALB/c mice, which were treated with 2%, 5%, 7.5% or 12.5% of NAM (*w*/*v*) for a period of seven weeks (Figure 2A). Supplementation with NAM has an effective measure in the amelioration of the typical skin photoaging phenotypes, including erythema, wrinkles, and signs of extensive flaking (Figure 2B). NAM reduced the skin photoaging score (Figure 2C). HE staining showed that the photoaging mice exhibited increased keratinization of the epidermal layer, thickening of acanthocytes, and varying degrees of inflammatory cell infiltration, whereas NAM administration resulted in significant improvement (Figure 2D). In addition, the average epidermal thickness of photoaging mice was 81.4 ± 3.41 μm compared to 17.7 ± 7.63 μm in the normal mice. The average epidermal thickness of the mice skin treated with 2%, 5%, 7.5% or 12.5% NAM (*w*/*v*) for photoaging treatment was found to be 26.51 ± 5.33 μm, 28.13 ± 5.64 μm, 36.07 ± 7.80 μm, and 35.82 ± 8.10 μm, respectively (Figure 2E). The findings of the study indicate that NAM ameliorates the skin photoaging of mice.

### 3.3. Supplementing with NAM Modulates the NAD^+^ Metabolic Network in Photoaging Mice

**Figure 3:**
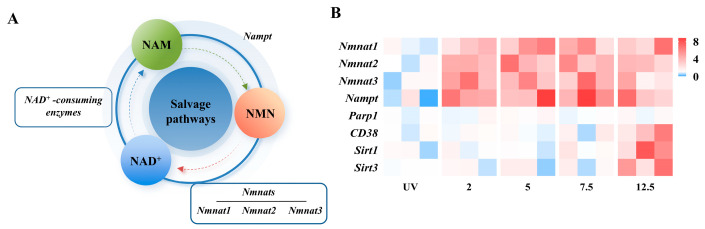
NAD^+^ synthesis-related genes are down-regulated during skin aging. (**A**) Schematic of the NAD^+^ salvage pathway. (**B**) The mRNA levels of the salvage pathway enzymes and NAD-consuming enzymes in the skin of aged mice. The heat map illustrates the results of RT-qPCR analysis.

NAM is first converted to NMN by NAMPT—the principal rate-limiting enzyme of this route—followed by NMNAT-mediated adenylation to yield NAD^+^ [8]. This pathway recycles NAM that is generated from NAD^+^-consuming reactions—such as those catalyzed by sirtuins—and thus constitutes a closed metabolic cycle (Figure 3A). To evaluate the impact of NAM on the salvage arm of the NAD^+^ biosynthetic pathway in the skin tissues of photoaging mice, we quantified the mRNA levels of the key enzymes involved in this route. We found that the mRNA levels of salvage-pathway enzymes including *Nampt* and *Nmnat1*, *Nmnat2*, and *Nmnat3* were increased in photoaging mice supplementation with 2–12.5% NAM (*w*/*v*) (Figure 3B). In addition, we found that the mRNA levels of the NAD^+^-consuming enzymes *Cd38 and* the sirtuins *Sirt1* and *Sirt3* were increased in photoaging mice supplementation with 12.5% NAM (*w*/*v*), but not the NAD^+^-consuming enzymes *Parp1* (Figure 3B). Collectively, these results reveal that NAM improves photoaging by regulating the expression the salvage-pathway enzymes and NAD^+^ consumers.

### 3.4. NAM Restores Skin Barrier Homeostasis of Photoaging Mice

**Figure 4:**
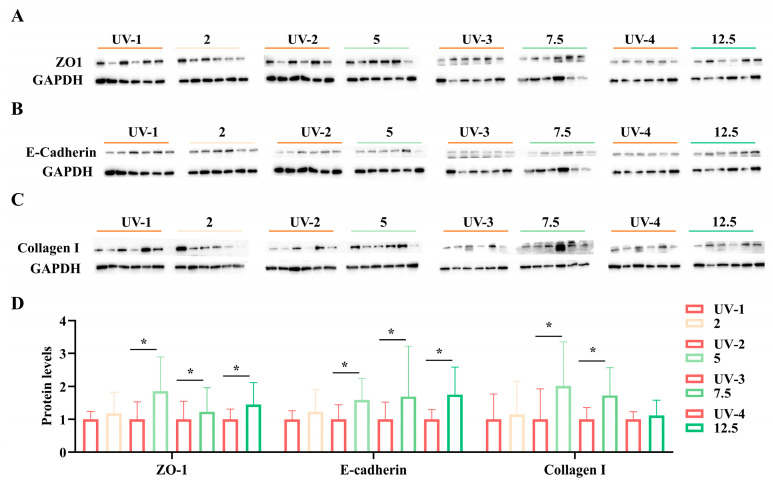
NAM improves the collagen and the skin barrier damage in photoaging mice. (**A**–**C**) The protein levels of ZO1 (**A**), E-cadherin (**B**), and collagen I (**C**) were determined by Western blot among UV, UV + 2% (*w*/*v*) NAM, UV + 5% (*w*/*v*) NAM, UV + 7.5% (*w*/*v*) NAM, and UV + 12.5% (*w*/*v*) NAM. (**D**) Quantification of the ratio of ZO1, E-cadherin and collagen I to GAPDH. These results are means ± SD of three experiments. * *p* < 0.05. *p* values (**D**) were calculated using a two-tailed unpaired *t*-test.

To evaluate the effect of NAM on skin barrier homeostasis in photoaging mice, we assessed the levels of three proteins essential for maintaining skin structure, function, and health: the tight junction protein E-cadherin, the adherens junction protein ZO1, and collagen I [25,26,27]. We found that supplementation with NAM restored the expression of both ZO1 (Figure 4A,D) and E-cadherin (Figure 4B,D) in photoaging mice, and the optimal concentration of NAM (*w*/*v*) for this effect was determined to be 5–12.5%. Furthermore, we observed that NAM reversed the decrease in collagen I expression in the photoaging mice (Figure 4C,D), and the optimal concentration of NAM (*w*/*v*) as well as 5–7.5%. It is evident that the NAM restored the expression of skin barrier-related proteins and type I collagen in photoaging mice.

### 3.5. NAM Inhibits the Expression of Cellular Senescence Biomarkers in Skin Photoaging

**Figure 5:**
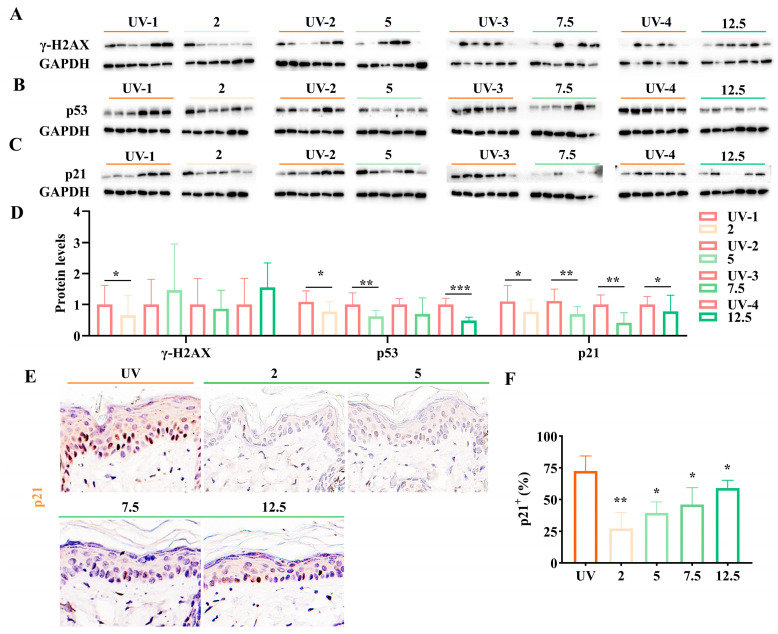
NAM inhibits the expression of cellular senescence biomarkers in photoaging mice. (**A**–**C**) The protein levels of γ-H2AX (**A**), p53 (**B**), and p21 (**C**) were determined by Western blot among UV, UV + 2% (*w*/*v*) NAM, UV + 5% (*w*/*v*) NAM, UV + 7.5% (*w*/*v*) NAM, and UV + 12.5% (*w*/*v*) NAM. (**D**) Quantification of the ratio of γ-H2AX, p53 and p21 to GAPDH. These results are means ± SD of three experiments. * *p* < 0.05, ** *p* < 0.01, *** *p* < 0.001. (**E**) Representative immunohistochemical images of p21. (**F**) The p21 positive cells were quantified. These results are means ± SD of three experiments. * *p* < 0.05, ** *p* < 0.01. *p* values (**D**) were calculated using a two-tailed unpaired *t*-test. *p* values (**F**) were calculated using one-way ANOVA followed by Tukey’s test in multiple groups.

We tested the expression of γ-H2AX, p53 and p21, which have been identified as indicators of photoaging as well as biomarkers of cellular senescence [28]. We found that the protein levels of the γ-H2AX were reduced in photoaging mice supplementation with 2% NAM (*w*/*v*) compared to the photoaging (UV) mice (Figure 5A,D). Furthermore, we found that the protein levels of p53 and p21 were reduced in photoaging mice supplementation with NAM compared to the photoaging mice (Figure 5B–D), and this effective concentration of NAM (*w*/*v*) is 2–12.5%. Meanwhile, we confirmed the result of p21 by immunohistochemistry staining (Figure 5E,F). These findings indicate that NAM plays a significant role in delaying cellular senescence of skin photoaging in mice by inhibiting the p53-p21 senescence pathway.

### 3.6. NAM Suppress the Expression of SASP Factors in Skin Photoaging

**Figure 6:**
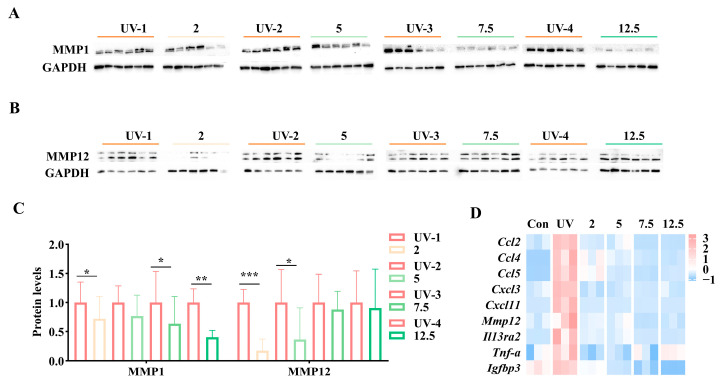
NAM suppressed the expression of SASP factors in photoaging mice. (**A**,**B**) The protein levels of MMP1 (**A**) and MMP12 (**B**) were determined by Western blot among UV, UV + 2% (*w*/*v*) NAM, UV + 5% (*w*/*v*) NAM, UV + 7.5% (*w*/*v*) NAM, and UV + 12.5% (*w*/*v*) NAM. (**C**) Quantification of the ratio of MMP1 and MMP12 to GAPDH. These results are means ± SD of three experiments. * *p* < 0.05, ** *p* < 0.01, *** *p* < 0.001. *p* values were calculated using a two-tailed unpaired *t*-test. (**D**) The mRNA levels of SASP factors were reduced in the skin of photoaging mice treated with NAM. The heat map illustrates the results of RT-qPCR analysis.

The secretion of a multitude of chemokines, cytokines, growth factors and proteases by senescent cells is recognized as a senescence-associated secretory phenotype (SASP) and is a hallmark of the cellular senescence [29]. By Western blot analysis, we found that the protein levels of matrix metalloproteinase MMP1 (Figure 6A,C) and MMP12 (Figure 6A,C) were significantly reduced in photoaging mice supplementation with 2% and 5% NAM (*w*/*v*).

Using qPCR, we found that the expression of the SASP factors was upregulated in skin photoaging, including cytokine/chemokines *Ccl2/4/5*, *Cxcl3/11*, matrix metalloproteinase *Mmp12*, proinflammatory factors *Tnf-α*, protease inhibitor *Igfbp3*, and Interleukin 13 receptor subunit A2 *Il13ra2* (Figure 6D). Moreover, treatment with 2%, 5%, 7.5% or 12.5% NAM (*w*/*v*) reduced the mRNA levels of these SASP factors in photoaging mice (Figure 6D). The findings indicate that NAM plays a significant role in suppressing of the expression of SASP factors of skin photoaging in mice.

## 4. Discussion

This study demonstrates that NAM treatment effectively ameliorated the hallmark features of photoaging in mice. Supplementation of NAM improves photoaging by modulating the NAD^+^ metabolic network. At the molecular level, these benefits were associated with the upregulation of epidermal barrier proteins (E-cadherin and ZO1) and type I collagen, alongside the downregulation of markers for DNA damage (γ-H2AX), cell cycle arrest/senescence (p53 and p21), and SASP factors (e.g., MMP1 and MMP12). Collectively, these findings indicated that NAM exerts its anti-photoaging effects by targeting multiple critical steps in the process: delaying cellular senescence and suppressing the SASP.

A decline in NAD^+^ levels occurs naturally with advancing age. A correlation between lower NAD^+^ levels and various chronic age-related diseases has been demonstrated [30,31]. It serves as a substrate or cofactor to mediate the activity of histone deacetylases and poly (ADP-ribose) polymerases [8]. These enzymes have been shown to regulate a variety of biological processes, including DNA repair, chromatin remodeling, and stress responses. The supplementation of the precursors nicotinamide riboside (NR) or nicotinamide mononucleotide (NMN) has been observed to enhance NAD^+^ levels, potentially mitigating age-related physiological degeneration [32,33]. However, the research has indicated that NAM prevents photoaging in two-dimensional human primary keratinocyte cultures and three-dimensional organotypic epidermal models [20]. The study demonstrates that NAM prevents photoaging by facilitating DNA repair while maintaining energy metabolism in epidermal cells [20]. Our study provides in vivo evidence to support that NAM plays a role in mitigating photoaging in mouse. We found that NAM inhibits the expression of cellular senescence biomarkers (γ-H2AX, p53 and p21) in photoaging mice. Consistent with previous studies [27], NAM enhances the repair of UV-induced DNA damage. This result can be attributed to the function of NAM as a precursor of NAD^+^, the substrate for Poly (ADP-ribose) polymerase-1 (PARP-1) catalyzed DNA repair [27,28]. Thus, these results indicate that NAM enhances genomic maintenance and retards the activation of the p53-p21 senescence pathway in skin photoaging. However, whether NAM exerts its anti-photoaging effects through NAD^+^ remains to be determined.

A recent study reported that oral supplementation with NR or NMN nearly doubled circulating NAD^+^ levels in human participants, whereas NAM showed no significant elevation relative to placebo [34]. This apparent discrepancy arises may be due to that NR and NMN are converted to NA via gut microbial metabolism, which then steadily drives systemic NAD^+^ repletion. In contrast, NAM undergoes rapid uptake and is promptly metabolized, resulting in a transient rather than sustained increase in blood NAD^+^. However, compared with other NAD^+^ precursors, such as NMN (334.22 g/mol) and NR (255.25 g/mol), NAM (122.12 g/mol) has the smallest molecular mass, which, together with its high aqueous solubility and neutral charge at physiological pH, favors passive transdermal permeation. In this study we found that supplementation of NAM improves photoaging by modulating the NAD^+^ metabolic network.

SASPs consist of pro-inflammatory cytokines, growth factors, insulin-like growth factor binding protein, chemokines and MMPs [35]. The expression of SASP factors including IL-6, TNF, IL-1α, IL-1β, CXCL8, CXCL10, and MMPs was significantly increased in skin photoaging [1,35,36]. We found that the expression of the SASP factors was upregulated in skin photoaging, including CCL2/4/5, CXCL3/11, MMP1/12, TNF-α, IGFBP3, and IL13RA2. Furthermore, NAM significantly downregulated the expression of these SASP factors in skin photoaging. This broader suppression is consistent with the documented ability of NAM to inhibit SASP in models of alcoholic liver disease [13]. Our data further show that NAM significantly downregulated the expression of the matrix-degrading enzymes MMP-1 and MMP-12. Since MMP-1 is the major collagenase for type I collagen [37,38,39] and MMP-12 is a critical elastase [40,41], their suppression directly underpins the concomitant increase in collagen I levels we observed. These results indicate that NAM plays a significant role in suppressing the expression of SASP factors of skin photoaging.

Interestingly, while NAM improved epidermal thickness at all concentrations (2–12.5%), the intermediate concentrations (5% and 7.5%) appeared more effective for certain protein markers, such as p21 and collagen I, compared to the highest concentration (12.5%). Research indicates that the beneficial effects of topical NAM on skin barrier function and pigmentation have been shown to plateau at concentrations above 5% [42]. In addition, the mRNA levels of the salvage-pathway enzymes *Nampt* and *Nmnats* did not increase in photoaging mice supplemented with the highest concentration of NAM compared to those supplemented with the low or intermediate concentration of NAM. The beneficial effects of NAM on skin barrier proteins and the cellular senescence biomarkers may reach a plateau at approximately 5–7.5%, beyond which additional NAM provides no further benefit.

Taken together, our study reveals that NAM counteracts photoaging through a coordinated mechanism: alleviating senescence drivers, quenching the downstream SASP response, and ultimately reinstating skin barrier and dermal matrix homeostasis. The results obtained demonstrate that NAM is a promising multitargeted therapeutic candidate with the potential to ameliorate skin photoaging.

## Data Availability

All of the above data sets are from GEO public database (https://www.ncbi.nlm.nih.gov/geo/, accession date: 22 July 2025). We collected two data sets of mouse skin tissues, including GSE296578 and GSE279221. For single-cell transcriptomic data, we collected GSE274955.

## Supplementary Materials

The following supporting information can be downloaded at: https://www.mdpi.com/article/10.3390/cimb48070661/s1.

## Abbreviations

The following abbreviations are used in this manuscript:

NAM	Nicotinamide
NAD^+^	Nicotinamide adenine dinucleotide
NA	Nicotinic acid
NR	Nicotinamide nucleoside
QA	quinolinic acid
SASP	Senescence-associated secretory phenotype
UV	Ultraviolet
ROS	Reactive oxygen species
ECM	extracellular matrix

## References

[1] Kaltchenko M.V., Chien A.L. (2025). Photoaging: Current Concepts on Molecular Mechanisms, Prevention, and Treatment. Am. J. Clin. Dermatol..

[2] Fisher G.J., Kang S., Varani J., Bata-Csorgo Z., Wan Y., Datta S., Voorhees J.J. (2002). Mechanisms of photoaging and chronological skin aging. Arch. Dermatol..

[3] Ananthaswamy H.N., Pierceall W.E. (1990). Molecular mechanisms of ultraviolet radiation carcinogenesis. Photochem. Photobiol..

[4] Zhang Z., Tan R., Xiong Z., Feng Y., Chen L. (2025). Dysregulation of autophagy during photoaging reduce oxidative stress and inflammatory damage caused by UV. Front. Pharmacol..

[5] Jeremian R., Malinowski A., Lytvyn Y., Georgakopoulos J.R., Muntyanu A., Mufti A., Lefrancois P., Yeung J., Litvinov I.V. (2024). Skin photoageing following sun exposure is associated with decreased epigenetic and biologic age, and correlates with basal cell carcinoma phenotype. Br. J. Dermatol..

[6] Pei Z., Liang F., Wang X., Li H. (2026). NAD(+) as a central metabolic hub Regulating the hallmarks of aging: Mechanisms and therapeutic implications. Mech. Ageing Dev..

[7] Vinten K.T., Tretowicz M.M., Coskun E., van Weeghel M., Canto C., Zapata-Perez R., Janssens G.E., Houtkooper R.H. (2025). NAD(+) precursor supplementation in human ageing: Clinical evidence and challenges. Nat. Metab..

[8] Covarrubias A.J., Perrone R., Grozio A., Verdin E. (2021). NAD(+) metabolism and its roles in cellular processes during ageing. Nat. Rev. Mol. Cell Biol..

[9] Lautrup S., Hou Y., Fang E.F., Bohr V.A. (2024). Roles of NAD(+) in Health and Aging. Cold Spring Harb. Perspect. Med..

[10] Rajman L., Chwalek K., Sinclair D.A. (2018). Therapeutic Potential of NAD-Boosting Molecules: The In Vivo Evidence. Cell Metab..

[11] Zynda W., Ruczaj A., Galicka A. (2025). Natural Compounds with Beneficial Effects on Skin Collagen Type I and Mechanisms of Their Action. Antioxidants.

[12] Ong R.R., Goh C.F. (2024). Niacinamide: A review on dermal delivery strategies and clinical evidence. Drug Deliv. Transl. Res..

[13] Gold N.M., Ding Q., Yang Y., Pu S., Cao W., Ge X., Yang P., Okeke M.N., Nisar A., Pan Y. (2025). Therapeutic potential of nicotinamide and ABT263 in alcohol-associated liver disease through targeting cellular senescence. MedComm.

[14] Kang H.T., Lee H.I., Hwang E.S. (2006). Nicotinamide extends replicative lifespan of human cells. Aging Cell.

[15] Marques C., Hadjab F., Porcello A., Lourenco K., Scaletta C., Abdel-Sayed P., Hirt-Burri N., Applegate L.A., Laurent A. (2024). Mechanistic Insights into the Multiple Functions of Niacinamide: Therapeutic Implications and Cosmeceutical Applications in Functional Skincare Products. Antioxidants.

[16] Draelos Z., Bogdanowicz P., Saurat J.H. (2024). Top weapons in skin aging and actives to target the consequences of skin cell senescence. J. Eur. Acad. Dermatol. Venereol..

[17] Bissett D.L., Oblong J.E., Berge C.A. (2005). Niacinamide: A B vitamin that improves aging facial skin appearance. Dermatol. Surg..

[18] Bissett D.L., Miyamoto K., Sun P., Li J., Berge C.A. (2004). Topical niacinamide reduces yellowing, wrinkling, red blotchiness, and hyperpigmented spots in aging facial skin. Int. J. Cosmet. Sci..

[19] Boo Y.C. (2021). Mechanistic Basis and Clinical Evidence for the Applications of Nicotinamide (Niacinamide) to Control Skin Aging and Pigmentation. Antioxidants.

[20] Tan C.Y.R., Tan C.L., Chin T., Morenc M., Ho C.Y., Rovito H.A., Quek L.S., Soon A.L., Lim J.S.Y., Dreesen O. (2022). Nicotinamide Prevents UVB- and Oxidative Stress–Induced Photoaging in Human Primary Keratinocytes. J. Investig. Dermatol..

[21] Zheng S., Deng R., Huang G., Ou Z., Shen Z. (2024). Screening the active ingredients of plants via molecular docking technology and evaluating their ability to reduce skin photoaging. Biogerontology.

[22] Subramanian A., Tamayo P., Mootha V.K., Mukherjee S., Ebert B.L., Gillette M.A., Paulovich A., Pomeroy S.L., Golub T.R., Lander E.S. (2005). Gene set enrichment analysis: A knowledge-based approach for interpreting genome-wide expression profiles. Proc. Natl. Acad. Sci. USA.

[23] Li L. (2025). Human skin rejuvenation via mRNA. J. Investig. Dermatol..

[24] Kuehne A., Hildebrand J., Soehle J., Wenck H., Terstegen L., Gallinat S., Knott A., Winnefeld M., Zamboni N. (2017). An integrative metabolomics and transcriptomics study to identify metabolic alterations in aged skin of humans in vivo. BMC Genom..

[25] Kroemer G., Maier A.B., Cuervo A.M., Gladyshev V.N., Ferrucci L., Gorbunova V., Kennedy B.K., Rando T.A., Seluanov A., Sierra F. (2025). From geroscience to precision geromedicine: Understanding and managing aging. Cell.

[26] Shao Q., Wang Z., Li Y., Tang X., Li Z., Xia H., Wu Q., Chang R., Wu C., Meng T. (2025). Taurine Prevents Impairments in Skin Barrier Function and Dermal Collagen Synthesis Triggered by Sleep Deprivation-Induced Estrogen Circadian Rhythm Disruption. Cells.

[27] Celli A., Tu C.L., Lee E., Bikle D.D., Mauro T.M. (2021). Decreased Calcium-Sensing Receptor Expression Controls Calcium Signaling and Cell-To-Cell Adhesion Defects in Aged Skin. J. Investig. Dermatol..

[28] Wang A.S., Dreesen O. (2018). Biomarkers of Cellular Senescence and Skin Aging. Front. Genet..

[29] Zhang M., Lin Y., Han Z., Huang X., Zhou S., Wang S., Zhou Y., Han X., Chen H. (2024). Exploring mechanisms of skin aging: Insights for clinical treatment. Front. Immunol..

[30] Camacho-Pereira J., Tarrago M.G., Chini C.C.S., Nin V., Escande C., Warner G.M., Puranik A.S., Schoon R.A., Reid J.M., Galina A. (2016). CD38 Dictates Age-Related NAD Decline and Mitochondrial Dysfunction through an SIRT3-Dependent Mechanism. Cell Metab..

[31] Schultz M.B., Sinclair D.A. (2016). Why NAD(+) Declines during Aging: It’s Destroyed. Cell Metab..

[32] Yoshino J., Imai S. (2011). Mitochondrial SIRT3: A new potential therapeutic target for metabolic syndrome. Mol. Cell.

[33] Bita A., Scorei I.R., Ciocilteu M.V., Nicolaescu O.E., Pirvu A.S., Bejenaru L.E., Rau G., Bejenaru C., Radu A., Neamtu J. (2023). Nicotinamide Riboside, a Promising Vitamin B(3) Derivative for Healthy Aging and Longevity: Current Research and Perspectives. Molecules.

[34] Christen S., Redeuil K., Goulet L., Giner M.P., Breton I., Rota R., Frezal A., Nazari A., Van den Abbeele P., Godin J.P. (2026). The differential impact of three different NAD(+) boosters on circulatory NAD and microbial metabolism in humans. Nat. Metab..

[35] Wang B., Han J., Elisseeff J.H., Demaria M. (2024). The senescence-associated secretory phenotype and its physiological and pathological implications. Nat. Rev. Mol. Cell Biol..

[36] Sun J.M., Liu Y.X., Liu Y.D., Ho C.K., Tsai Y.T., Wen D.S., Huang L., Zheng D.N., Gao Y., Zhang Y.F. (2024). Salvianolic acid B protects against UVB-induced skin aging via activation of NRF2. Phytomedicine.

[37] Varani J., Dame M.K., Rittie L., Fligiel S.E., Kang S., Fisher G.J., Voorhees J.J. (2006). Decreased collagen production in chronologically aged skin: Roles of age-dependent alteration in fibroblast function and defective mechanical stimulation. Am. J. Pathol..

[38] Sarkar S.K., Marmer B., Goldberg G., Neuman K.C. (2012). Single-molecule tracking of collagenase on native type I collagen fibrils reveals degradation mechanism. Curr. Biol..

[39] Trentini M., Zanolla I., Zanotti F., Tiengo E., Licastro D., Dal Monego S., Lovatti L., Zavan B. (2022). Apple Derived Exosomes Improve Collagen Type I Production and Decrease MMPs during Aging of the Skin through Downregulation of the NF-kappaB Pathway as Mode of Action. Cells.

[40] Hautamaki R.D., Kobayashi D.K., Senior R.M., Shapiro S.D. (1997). Requirement for macrophage elastase for cigarette smoke-induced emphysema in mice. Science.

[41] Chen Z., Seo J.Y., Kim Y.K., Lee S.R., Kim K.H., Cho K.H., Eun H.C., Chung J.H. (2005). Heat modulation of tropoelastin, fibrillin-1, and matrix metalloproteinase-12 in human skin in vivo. J. Investig. Dermatol..

[42] Greatens A., Hakozaki T., Koshoffer A., Epstein H., Schwemberger S., Babcock G., Bissett D., Takiwaki H., Arase S., Wickett R.R. (2005). Effective inhibition of melanosome transfer to keratinocytes by lectins and niacinamide is reversible. Exp. Dermatol..

