# Supplementary material for: Nicotinamide Improves Skin Photoaging in Mice by Delaying Cellular Senescence and Suppressing the Senescence-Associated Secretory Phenotype
Source: Curr Issues Mol Biol. 2026 Jun 27;48(7):661. doi: 10.3390/cimb48070661 (PMC13406687; doi:10.3390/cimb48070661)
Supplement: Supplementary file 1 [file cimb-48-00661-s001.zip › cimb-4368661-supplementary.pdf]

Supplementary Information

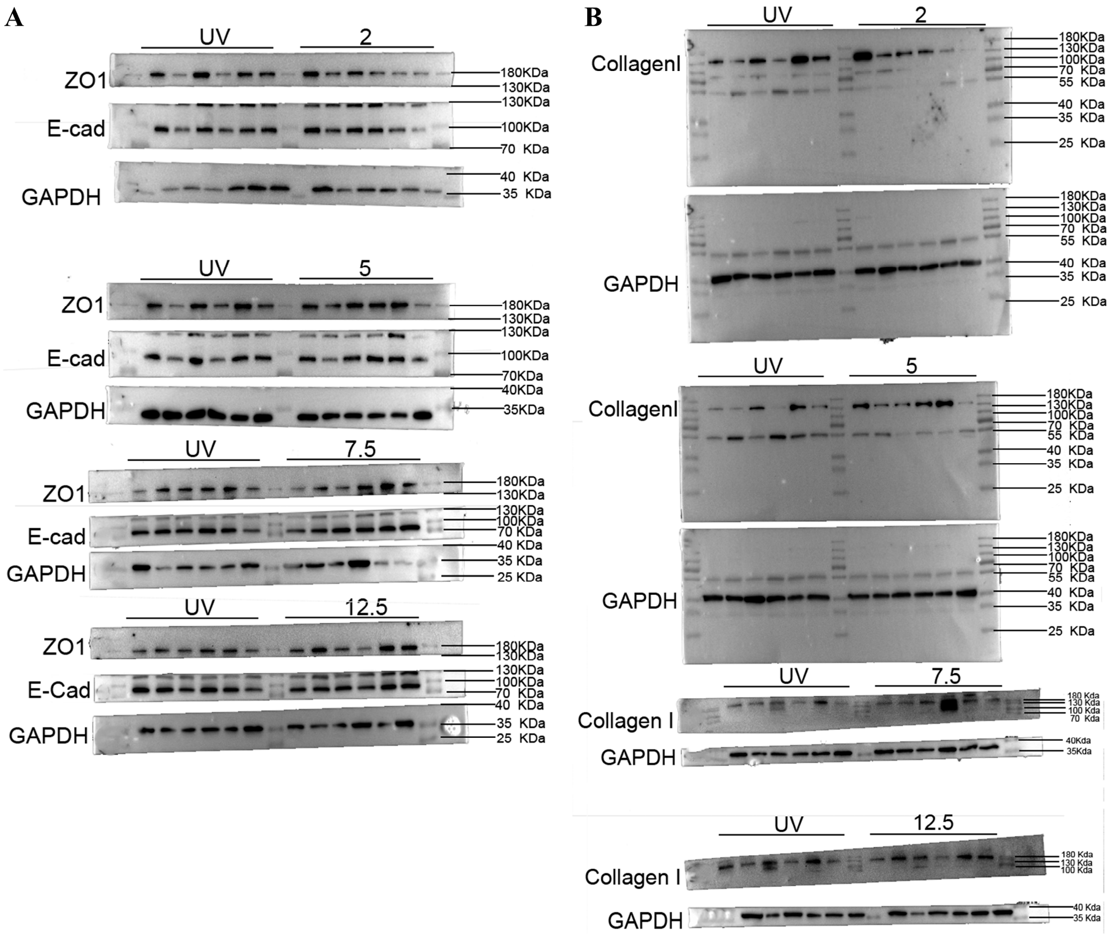

Figure S1 Full scans of the Western blot image in Figure 3.

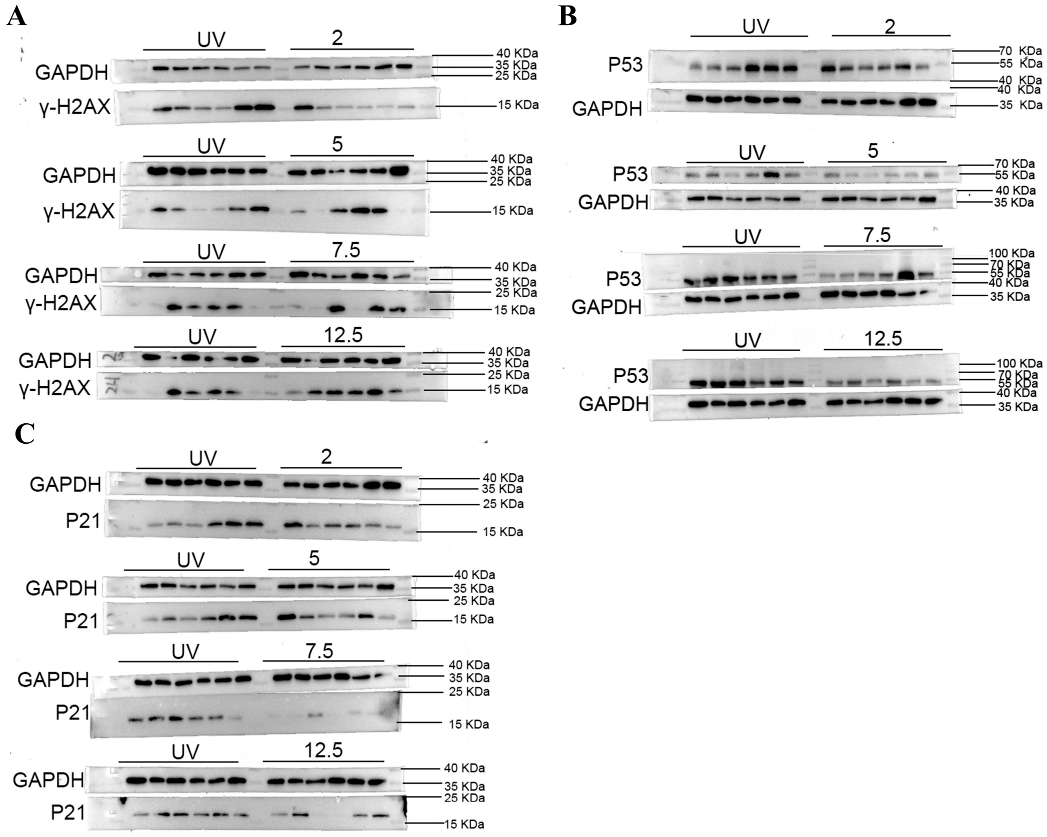

Figure S2 Full scans of the Western blot image in Figure 4.

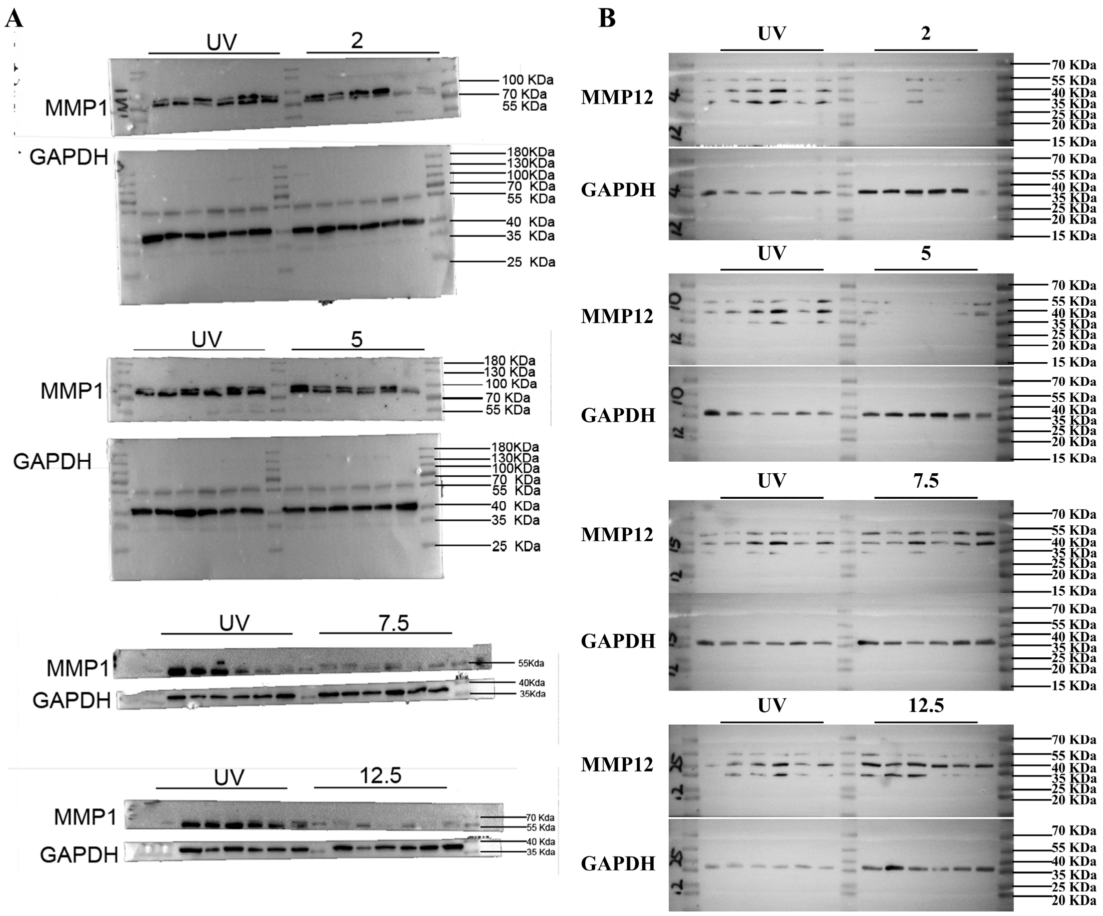

Figure S3 Full scans of the Western blot image in Figure 5.

Table S1. The intensity of UV exposure varied of photoaging mouse.

week	UVB (mJ/cm2)	UVB (s)	UVA (mJ/cm2)	UVA (s)
1	70	34	1370	34
2	105	50	2055	50
3	140	67	2740	67
4	175	84	3425	84
5	210	100	4110	100
6	245	117	4795	117
7	280	134	5480	134
8	315	150	6165	150
9	350	167	6850	167

Table S2. Scoring rules for photoaging mice.

Grade	Skin features
1	Smooth skin; no wrinkles or laxity.
2	Smooth skin with small longitudinal wrinkles.
3	The skin is rough with a little bit shallow wrinkles.
4	The skin is obviously rough and loose, shallow wrinkles across the dorsal skin.
5	The skin is rough and thick, with erythema, skin lesions, desquamation and a few deep wrinkles.
6	The skin is rough and thick, more desquamation, the skin color is deepened, and the deep horizontal wrinkles are increased.

Table S3. The sequence of primers for qPCR.

Gene name		Sequence (5'->3')
Ccl2	F	CCTGCTGTTCACAGTTGCC
	R	ATTGGGATCATCTTGCTGGT
Ccl4	F	GCCCTCTCTCTCCTCTTGCT
	R	GTCTGCCTCTTTTGGTCAGG
Ccl5	F	ATATGGCTCGGACACCACTC
	R	TTCGAGTGACAAACACGACTG
Cxcl3	F	CCAGACAGAAGTCATAGCCAC
	R	CTTCATCATGGTGAGGGGCTT
Cxcl11	F	ACAGGAAGGTCACAGCCATAG
	R	AGCTTTCTCGATCTCTGCCA
Mmp12	F	TTTGCATTTGGAGCTCACGG
	R	AGTCCACGTTTCTGCCTCAT
Il13ra2	F	GAGGACCCATTCCACCAAGG
	R	GAGTCTGGCCCTGTGTAACC
Tnf	F	GGACTAGCCAGGAGGGAGAA
	R	CGCGGATCATGCTTTCTGTG
Igfbp3	F	GTTCCATCCACTCCATGCCA
	R	CGGCAGGGACCGTATTCTG
Nampt	F	AGCTGAACATCGAGCAGGAC
	R	GGCCTTTGGGATTTGGCATC
Nmnat1	F	GTAAGCAACCCACCGAGGTC
	R	CCCACGTATCCACTTCCACC
Nmnat2	F	TGGATCAGTGCGAGAGACCT
	R	CAACGCTGACTCCCGAACA
Nmnat3	F	TAGCCCCACGGTCACTTTTC
	R	ACCTGGTACCTTCTTTTAACTGACT
CD38	F	GAGAGCCTACCACGAAGCAC
	R	AAAATGGCTCACGGAGGGAC
Parp1	F	AAGGCGGAGAAGACATTGGG
	R	ACCATCTTCTTGGACAGGCG
Sirt1	F	CGGCTACCGAGGTCCATATAC
	R	AACATGGCTTGAGGGTCTGG
Sirt3	F	CGGGAGTGTTACAGGTGGGA
	R	AAAGGGCTTGGGGTTGTGAA
Gapdh	F	GCAGTGGCAAAGTGGAGATT
	R	GAATTTGCCGTGAGTGGAGT
